# Human serum albumin alters specific genes that can play a role in survival and persistence in ***Acinetobacter baumannii***

**DOI:** 10.1038/s41598-018-33072-z

**Published:** 2018-10-03

**Authors:** Brettni Quinn, Nyah Rodman, Eugenio Jara, Jennifer S. Fernandez, Jasmine Martinez, German M. Traglia, Sabrina Montaña, Virginia Cantera, Kori Place, Robert A. Bonomo, Andres Iriarte, María Soledad Ramírez

**Affiliations:** 1 Center for Applied Biotechnology Studies, Department of Biological Science, College of Natural Sciences and Mathematics, California State University Fullerton, Fullerton, California, USA; 2 Área Genética, Facultad de Veterinaria, Universidad de la República, Montevideo, Uruguay; 3 Laboratorio de Bacteriología Clínica, Departamento de Bioquímica Clínica, Hospital de Clínicas José de San Martín, Facultad de Farmacia y Bioquímica, Buenos Aires, Argentina; 4 Instituto de Microbiología y Parasitología Médica (IMPaM, UBA-CONICET), Facultad de Medicina, Universidad de Buenos, Aires, Argentina; 5 Medical Service and GRECC, Louis Stokes Cleveland Department of Veterans Affairs Medical Center, Cleveland, Ohio, USA; 6 Departments of Medicine, Pharmacology, Molecular Biology and Microbiology, Biochemistry, Proteomics and Bioinformatics, Case Western Reserve University School of Medicine, Cleveland, Ohio, USA; 7 CWRU-Cleveland VAMC Center for Antimicrobial Resistance and Epidemiology (Case VA CARES), Cleveland, Ohio, USA; 8 Laboratorio de Biología Computacional, Dpto. de Desarrollo Biotecnológico, Instituto de Higiene, Facultad de Medicina, UdelaR, Montevideo, Uruguay

## Abstract

In the past few decades *Acinetobacter baumannii* has emerged as a notorious nosocomial pathogen because of its ability to acquire genetic material and persist in extreme environments. Recently, human serum albumin (HSA) was shown to significantly increase natural transformation frequency in *A. baumannii*. This observation led us to perform transcriptomic analysis of strain A118 under HSA induction to identify genes that are altered by HSA. Our results revealed the statistically significant differential expression of 296 protein-coding genes, including those associated with motility, biofilm formation, metabolism, efflux pumps, capsule synthesis, and transcriptional regulation. Phenotypic analysis of these traits showed an increase in surface-associated motility, a decrease in biofilm formation, reduced activity of a citric acid cycle associated enzyme, and increased survival associated with zinc availability. Furthermore, the expression of genes known to play a role in pathogenicity and antibiotic resistance were altered. These genes included those associated with RND-type efflux pumps, the type VI secretion system, iron acquisition/metabolism, and ß-lactam resistance. Together, these results illustrate how human products, in particular HSA, may play a significant role in both survival and persistence of *A. baumannii*.

## Introduction

*A. baumannii* is a pathogen that manifests a multi-drug-resistant (MDR) phenotype and infects patients in the critical care unit. As a result, outcomes are poor and the cost of care escalates^1–3^. Last year, the World Health Organization published a list of 12 antibiotic-resistant “priority pathogens” in which *A. baumannii* was listed as the first priority one pathogen for antibiotic research and development^4^. Some intrinsic features, such as the bacterium’s ability to persist in the clinical setting even under desiccation, nutrient starvation, and high concentrations of antimicrobials have allowed *A. baumannii* to become a major hospital-acquired pathogens^5^. In addition, extreme genome plasticity combined with its ability to acquire resistance determinants, play a key role in the evolution of *A. baumannii*’s phenotype. Comparative genomic studies have shown high variability in *Acinetobacter’s* genome organization, as well as the presence of foreign DNA in their genomes, suggesting exogenous acquisition of genetic traits^6–9^.

Due to the shrinking number of effective antimicrobials to treat *A. baumannii* infections, it is critical to understand the mechanisms of *A. baumannii* pathogenesis. Known virulence factors in *A. baumannii*, such as OmpA, phospholipase D, lipopolysaccharides, and production of the K1 capsule, promote colonization and can damage the host^10^. These factors can contribute to the pathogen’s overall survival against host immune effectors, however, these are not the only strategies the microbe may use to promote persistence. The persistence state is an evolutionarily adaptive strategy indicated by a change in metabolic profile, latent growth patterns, and has been associated with expression of toxin/antitoxin systems^11,12^. Persistence phenotypes are environmentally modulated, therefore, it is important to address changes in bacterial physiology under stress^13^.

Environmental signals within hospital settings and the host have been previously shown to play a role in virulence, biofilm formation, DNA-acquisition, and metabolism in *A. baumannii*^11–16^. Studies addressing the response of *A. baumannii* to environmental stimuli, such as mucin, light, antibiotics, bile salts, etc., demonstrate that *A. baumannii* behavior is affected^11–14^. An increase in genes involved in biofilm formation, degradation of phenylacetic acid, metabolic pathways, and genes coding for the type VI secretion system (T6SS), were observed in *A. baumannii* ATCC 19606^T^ ans *A. baumannii* ATCC 17978 under mucin or blue light treatment^11,14^. Furthermore, growth in the presence of bile salts resulted in an increase in expression of genes associated with acid tolerance, quorum sensing, T6SS, and surface motility/biofilm, results that were supported by phenotypic assays analyzing biofilm formation and surface associated motility^12^. Interestingly, transcriptomic analysis of MDR *A. baumannii* under different antibiotic treatments showed that antibiotic treatment was associated with an increase in expression with genes associated with transposable elements^13^.

In addition, *in-vivo* studies where transcriptomic analysis was performed to address the response of *A. baumannii* during bacteremia showed a decreased expression in 557 protein-coding genes, while 329 showed an increased expression^17^. This analysis also showed that genes associated with the siderophore iron uptake cluster were greatly up regulated. In addition, they observed that genes related to metabolism, quorum sensing and biofilm formation were also affected, showing its ability to adapt to changing environments^17^.

Other studies, where transposon-directed insertion-sequencing was used, 89 mutants were identified that showed a reduced fitness during bacteremia and spleen colonization^18^.

Recently, Traglia *et al*. 2016, showed that albumin, the main protein in blood, and Ca^2+^ significantly enhance transformation frequency and increase expression levels of two competence genes (*comEA* and *pilQ*) in *A. baumannii* strains^16^. The role of human serum albumin (HSA) in the adaptation to host environments and virulence was observed in other human pathogens, such as *Staphylococcus aureus*, group G streptococci, *Bordetella pertussis*, and *Toxoplasma gondii* among others^19–23^. Considering our previous observation, as well as those of others studying the dynamics of gene expression of *A. baumannii* challenged by a variety of stimuli, we conducted a transcriptomic analysis of *A. baumannii* (A118 strain) under HSA treatment to better understand the role of this human protein on the survival and persistence of this pathogen^15^. Surprisingly, we found that 296 genes showed statistically significant differences in expression under HSA treatment. Genes involved in motility, biofilm formation, efflux pumps, metabolism, capsule synthesis, transcriptional regulation, antibiotic resistance and pathogenesis were identified. The data obtained serves as the foundation to study key phenotypes related with the pathogenesis of this pathogen.

## Results and Discussion

### Identification of *A. baumannii* genes altered during exposure to HSA

Considering our previous observations on the role of HSA as an inducer of transformation^15^, we searched for genes that were expressed differently under these conditions by using RNA-seq technology.

Our analysis showed that HSA significantly affects the expression of 296 coding genes, of which 111 and 185 coding genes have a False Discovery Rate (FDR adjusted *P*-value) of <0.05 and <0.1, respectively and were considered to search and confirm relevant phenotypes (See Supplementary Fig. S1). Among them, 23 genes exhibited an increase in expression (up regulation) while 273 genes exhibited a decrease in expression (down regulation) (See Supplementary Fig. S1).

One hundred and eighty-nine of the differentially expressed gene products had annotated biological functions, which allowed us to categorize them in categories based on their location in the cell, their biological function and their molecular function (See Supplementary Fig. S2).

### Change in the expression of genes involved in motility and persistence

**Figure 1:**
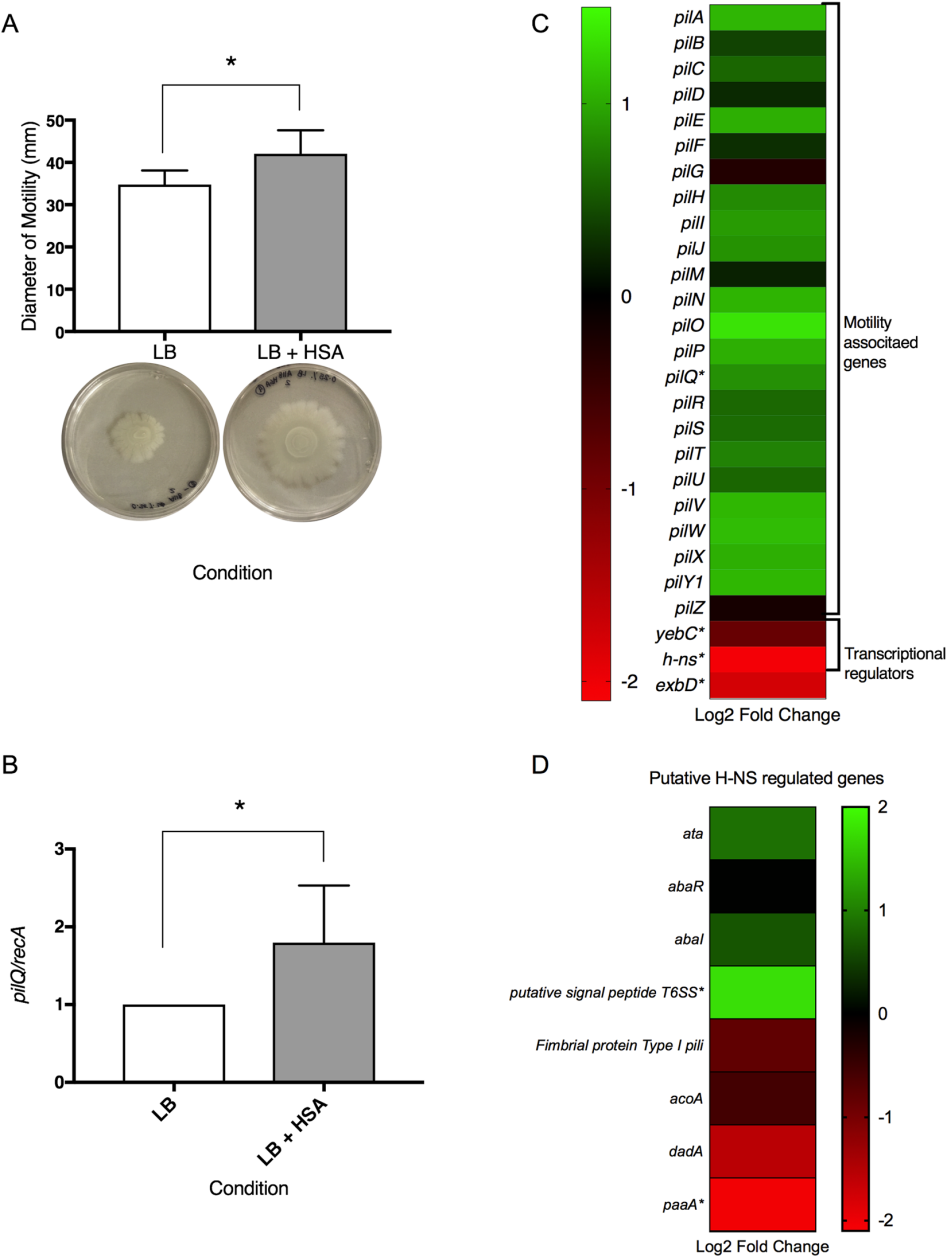
Phenotypic and genetic analysis of surface associated motility genes. (**A**) Motility assays resulted in a significant increase (*P-*value < 0.05) in the diameter of motility (mm) when grown in the presence of HSA. Cells grown with HSA had an average increase in diameter of motility of 7.28 mm. Experiments were performed in triplicate, with at least three technical replicates per biological replicate. Statistical analysis (Mann-Whitney test) was performed using GraphPad Prism (GraphPad software, San Diego, CA, USA), and a *P-*value < 0.05 was considered significant. (**B**) Quantitative PCR was performed to confirm the differential expression of *pilQ*. Results showed an increase in the expression of *pilQ* of 1.795-fold (*P*-value 0.0286). Experiments were performed in triplicate, with four technical replicates per biological replicate. Statistical analysis (Mann-Whitney test) was performed using GraphPad Prism (GraphPad software, San Diego, CA, USA), and a *P-*value < 0.05 was considered significant. (**C**) A heatmap outlining the differential expression of 30 genes associated with motility shows that the majority of motility associated genes are up regulated (green) while the three associated transcriptional regulators are down regulated (red). (**D**) A heatmap outlining the putative H-NS regulated genes shows variation in the differential expression of these genes.

Our transcriptomic analysis revealed four genes (*pilQ*, *yebC*, *h-ns, exbD*) significantly differentially expressed under HSA treatment that are involved in cell motility (see Supplementary Table S1). To examine the effect of HSA on surface associated motility, motility assays were performed. Our results showed an average 7.28 mm increase in motility diameter when cells were grown in the presence of HSA as compared to those grown in the control media (LB) (Fig. 1A).

As previously described, the type IV pilus is crucial in surface motility in *A. baumannii*^24^. During this process, the type IV pilus extends through a channel in the bacteria outer-membrane and binds to a surface in the environment. As retraction occurs, the pili remains bound and the bacterial cell is pulled, allowing it to move^25,26^. *pilQ*, a gene involved in surface motility and DNA acquisition, was expressed differentially under HSA induction. PilQ is the outer secretion protein found in type IV pili that allows double-stranded DNA to enter into the periplasm^27^. Previously, Traglia *et al*. 2016, showed that BSA can enhance transformation rates and induce the expression of the competence-related genes *comEA* and *pilQ*^16^. This result and the observations reported in Quinn *et al*. 2017, show that HSA has a singular and significant impact on transformation frequencies^15^. In addition, the expression of *pilQ* was confirmed to be statistically significant up regulated by qPCR analysis, which revealed a 1.80-fold increase in expression under HSA induction (Fig. 1B). After examining additional type IV pilus biogenesis and functions genes^28–30^, we found that 22 genes were also up regulated and only two genes were down regulated under HSA induction (Fig. 1C).

In addition, two transcriptional regulators (*yebC* and *h-ns*) that were expressed differently under HSA treatment were identified and may play a role in motility in *A. baumannii* under HSA induction (see Supplementary Table S1). NCBI BLAST and gene ontology analysis identified *yebC* as a putative transcriptional regulator that likely has sequence specific DNA binding activity. In *Pseudomonas aeruginosa*, the homologous gene product (61% identity) has been shown to negatively regulate quorum sensing^31,32^. Furthermore, it was recently shown that in *A. baumannii* the presence of a quorum quenching signal *aidA*, which inhibits quorum sensing, leads to a decrease in the expression of *pilT* and *adeB*^12^. As *pilT* is known to be essential for twitching motility, the decrease seen under quorum quenching conditions supports the hypothesis that *pilT* is up regulated under quorum sensing conditions^24^. Our transcriptomic analysis results identified *pilT* as up regulated with a log_2_ fold change of 0.77 (*P-*value = 0.46). In line with this, a slight increase in expression of *adeB* by log_2_ fold change of 0.4 under HSA induction was observed as well (*P-*value = 0.54). The gene *abaI*, involved in N-acyl homoserine lactone synthase activity, was also up regulated by a log_2_ fold change of 0.23 (*P-*value = 0.47) upon HSA exposition. Additionally, quorum quenching gene *aidA* and transcriptional regulator *yebC* were notably down regulated upon induction of HSA with a log_2_ fold change of −0.09 and −0.87 respectively (*P-*value > 0.05). Together, these results led us to hypothesize that these two genes may be negatively regulating quorum sensing genes in *A. baumannii* under HSA induction. Moreover, the decrease in expression of *aidA* and *yebC* could be in line with the increase in surface associated motility gene expression that was observed.

The other transcriptional regulator found significantly down regulated under HSA was the global transcriptional repressor *h-ns*, known to play a role in the expression of virulence traits^10^. Eijkelkamp *et al*. 2013, when investigating the genomic features of a hypermotile variant of the *A. baumannii* strain ATCC 17978, observed the disruption by an insertion sequences of the *h-ns* gene. This result led us to hypothesize that the decreased expression of *h-ns* (log_2_ fold change = −2.07) under HSA treatment could also explain in part the increased motility observed. H-NS was also observed to play a role in the regulation of gene expression of different virulence traits including the gene that codifies for the autotransporter Ata, the putative signal peptide of the Type VI secretion system, genes involved in biosynthesis of quorum-sensing signals, and genes of the phenylacetic acid degradation pathway^10^. Considering this data, we searched for those genes in our transcriptional analysis and we found the same tendency in the expression of the previous mentioned putative H-NS regulated genes (Fig. 1D).

The gene *exbD*, shown to be statistically significant and differentially expressed, is neither a pilus associated gene nor a transcriptional regulator. This gene caught our attention as it may play a role in Type IV pilus assembly and twitching motility^33^. This gene was identified as a biopolymer transporter by NCBI BLAST and is suspected to be a part of the ExbB-ExbD-TonB transport system in *A. baumannii*. While this system has been shown to play an important role in iron-acquisition in *A. baumannii*, its role in *E. coli* as flagellar motor proteins and its potential role in twitching motility in *P. aeruginosa* led us to propose that it could have a role in surface associated motility^34,35^. Interestingly, *exbD* is down regulated (log_2_ fold change = −1.77) under HSA induction, suggesting that further research is necessary to understand the complex relationship between this gene, motility and DNA uptake.

**Figure 2:**
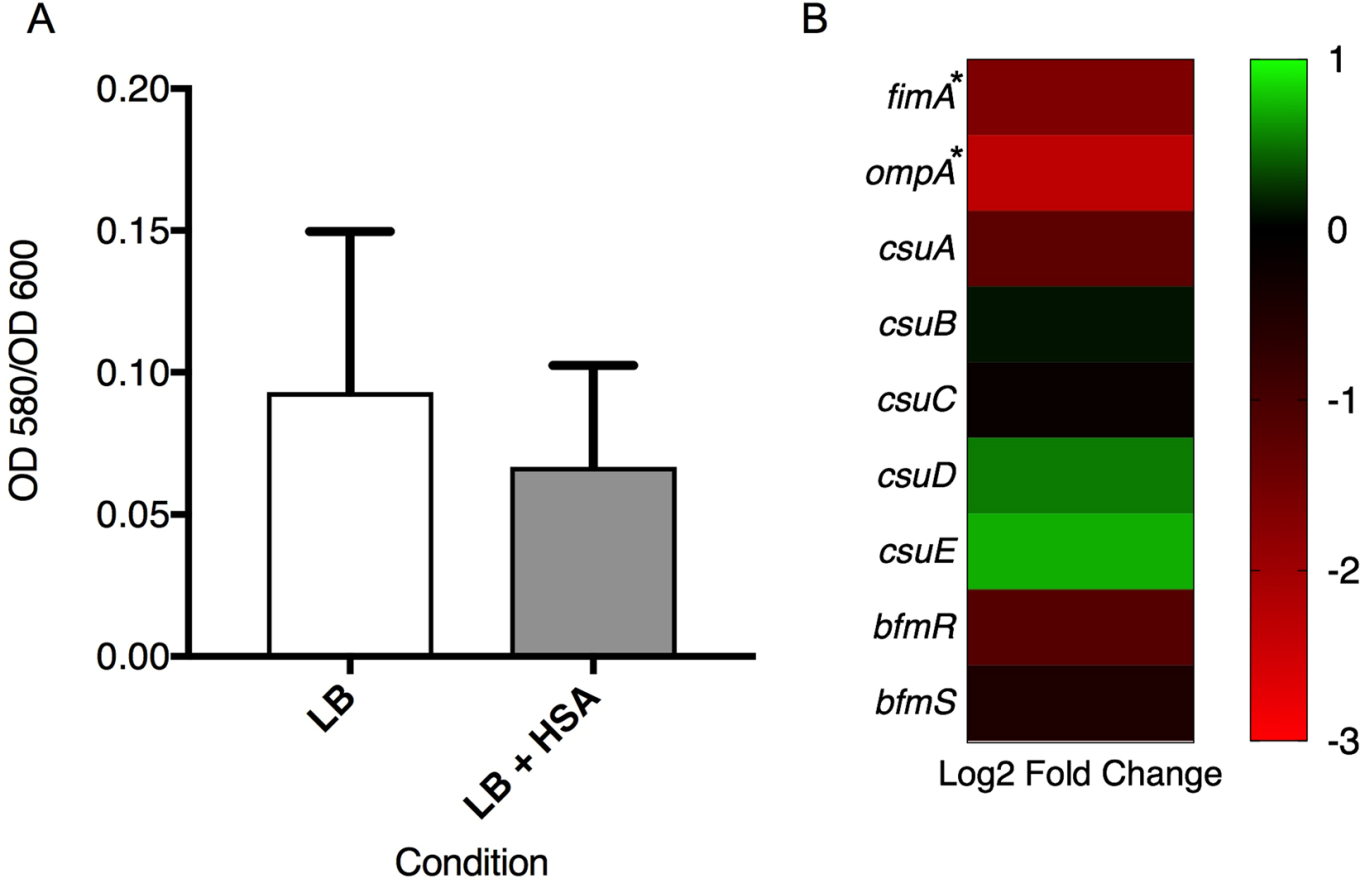
Phenotypic and genetic analysis of biofilm formation associated genes. (**A**) Biofilm assays performed with and without HSA show a decrease in biofilm formation (1.4-fold decrease), as represented by OD_580_/OD_600_, when grown in the presence of HSA. Experiments were performed in triplicate, with at least three technical replicates per biological replicate. Statistical analysis (Mann-Whitney test) was performed using GraphPad Prism (GraphPad software, San Diego, CA, USA), and a *P-*value < 0.05 was considered significant. (**B**) The associated heatmap outlines the differential expression of 9 genes associated with biofilm formation, including *fimA, ompA*, the *csu* operon and its regulator *bfmRS*.

Considering that biofilms play a key role in the survival and persistence of *A. baumannii* on biotic and abiotic surfaces, we searched for genes associated with biofilm formation in our transcriptomic analysis results^36^. We identified two genes, *fimA and ompA*, that were statistically differentially expressed under HSA induction. *fimA* gene was identified by NCBI BLAST as a Type I fimbrial protein and according to its associated gene ontology terms; this gene is hypothesized to play a role in cell adhesion and biofilm formation. In addition, *ompA* is known to play a role biofilm formation on plastic and for being essential for *A. baumannii* attachment to *Candida albicans* filaments and human alveolar epithelial cells^37^. Interestingly, these genes showed a log_2_ fold change of −1.64 and −2.32 which led us to test the ability of *A. baumannii* strain A118 to form biofilm in the presence or absence of HSA. Our result showed a 1.4-fold decrease (*P*-value = 0.31) in biofilm formation when cells were grown in the presence of HSA (Fig. 2A). Since it is well known that the *csu* operon is essential for *A. baumannii* biofilm formation, we searched in our transcriptomic analysis results for the level of expression of this operon under HSA treatment and found that while *csuA and csuC*, were down regulated, *csuBDE* were slightly up regulated (Fig. 2B) (see Supplementary Table S2)^36,38^. We also looked for the two-component regulatory system from *A. baumannii* that regulates biofilm formation, and we found that *bfmR* and *bfmS* were down regulated, which can in part explain the observed phenotype (Fig. 2B)^39^.

The ability to produce capsules is known to be an important virulence factor in several bacterial pathogens such as *Klebsiella pneumoniae, Streptococcus pneumoniae*, and *A. baumannii*^40^. In *A. baumannii*, capsule synthesis has been linked with the K locus, in which the genes present are responsible for activation of sugar precursors, capsule export, modifications of glycans etc.^41,42^. In a recent report, a subpopulation of *A. baumannii* cells with a thicker capsule and opaque appearance, were linked to an increase in antibiotic and disinfectant resistance as well as tolerance to desiccation^43^. The ability to switch between an opaque and translucent phenotype was linked to a master transcriptional regulator, which could cause the change between the virulent and non-virulent colonies, respectively. Possessing such a switch equips *A. baumannii* with the possibility to modulate its virulence in accordance with its environment.

**Figure 3:**
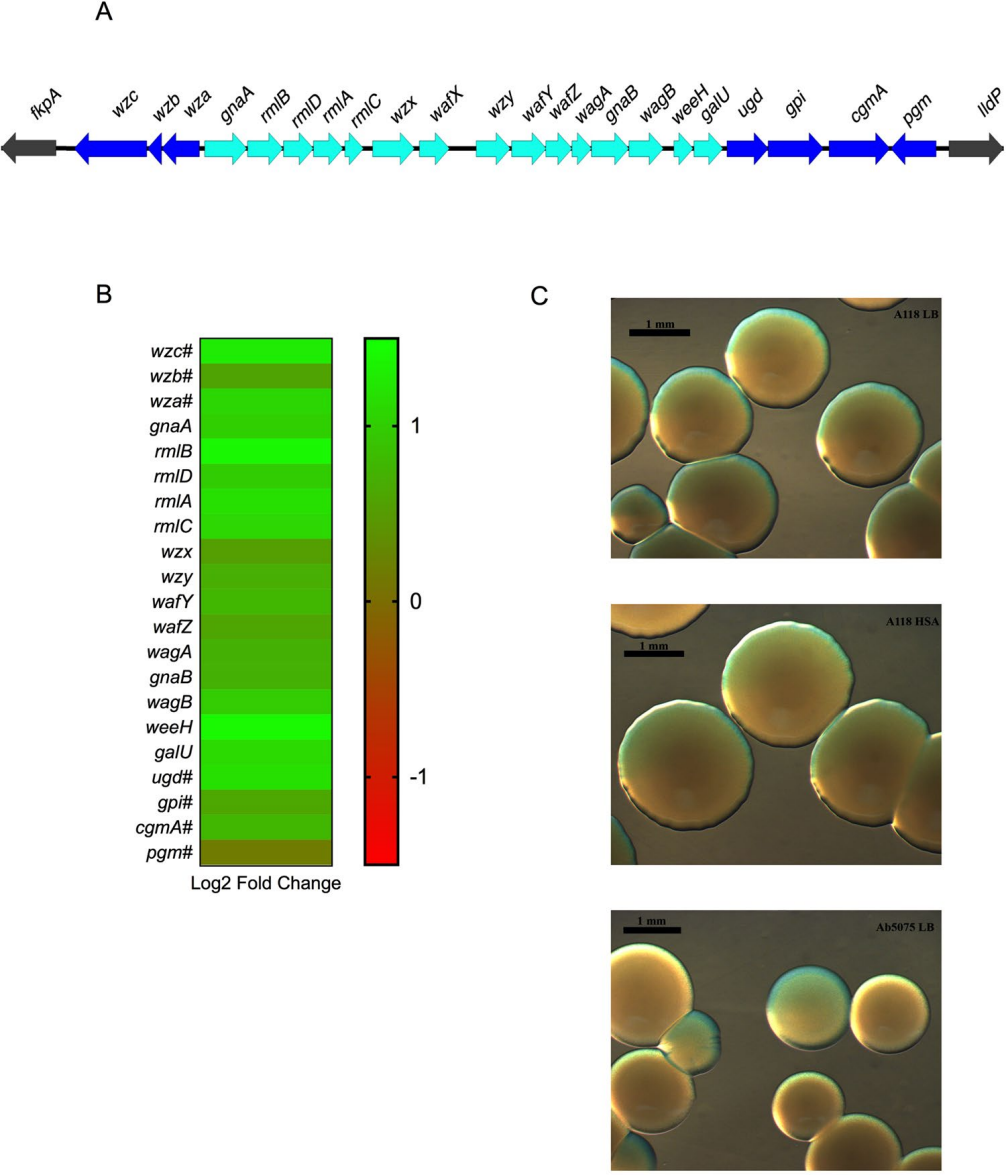
Phenotypic and genetic analysis of capsule synthesis genes. (**A**) The capsule synthesis operon as predicted for *A. baumannii* strain A118. (**B**) A heatmap outlining the differential expression of 21 genes that are members of the capsule synthesis operon. (**C**) Stereoscopic microscopy images showing capsule synthesis in A118 cells grown with or without HSA, A118 cells showed no difference in capsule synthesis when under HSA treatment. Ab5075 was used as a control.

According to our transcriptomic analysis, all 21 K locus genes present in A118 were up regulated under HSA induction, including *wzc* and *gpi*, which are statistically significantly differentially expressed, and are involved in the organization of capsule synthesis and carbohydrate biosynthesis, respectively (Fig. 3A,B and C)^42^. To explore the possibility of switching between opaque and translucent colonies, phase-contrast microscopy was used. A difference in capsule synthesis between growth in LB or LB + HSA was not seen and both conditions displayed an opaque phenotype (Fig. 3C). Interestingly, Tipton *et al*. 2015, linked an opaque phenotype to an increase in surface motility in the *A. baumannii* strain AB5075, which is consistent with the increase in motility under HSA treatment (Fig. 1A)^44^. Collectively, our transcriptomic results are in accordance with the phenotypic findings leading to the conclusion that *A. baumannii* can use capsule synthesis as a way to persist in the host.

**Figure 4:**
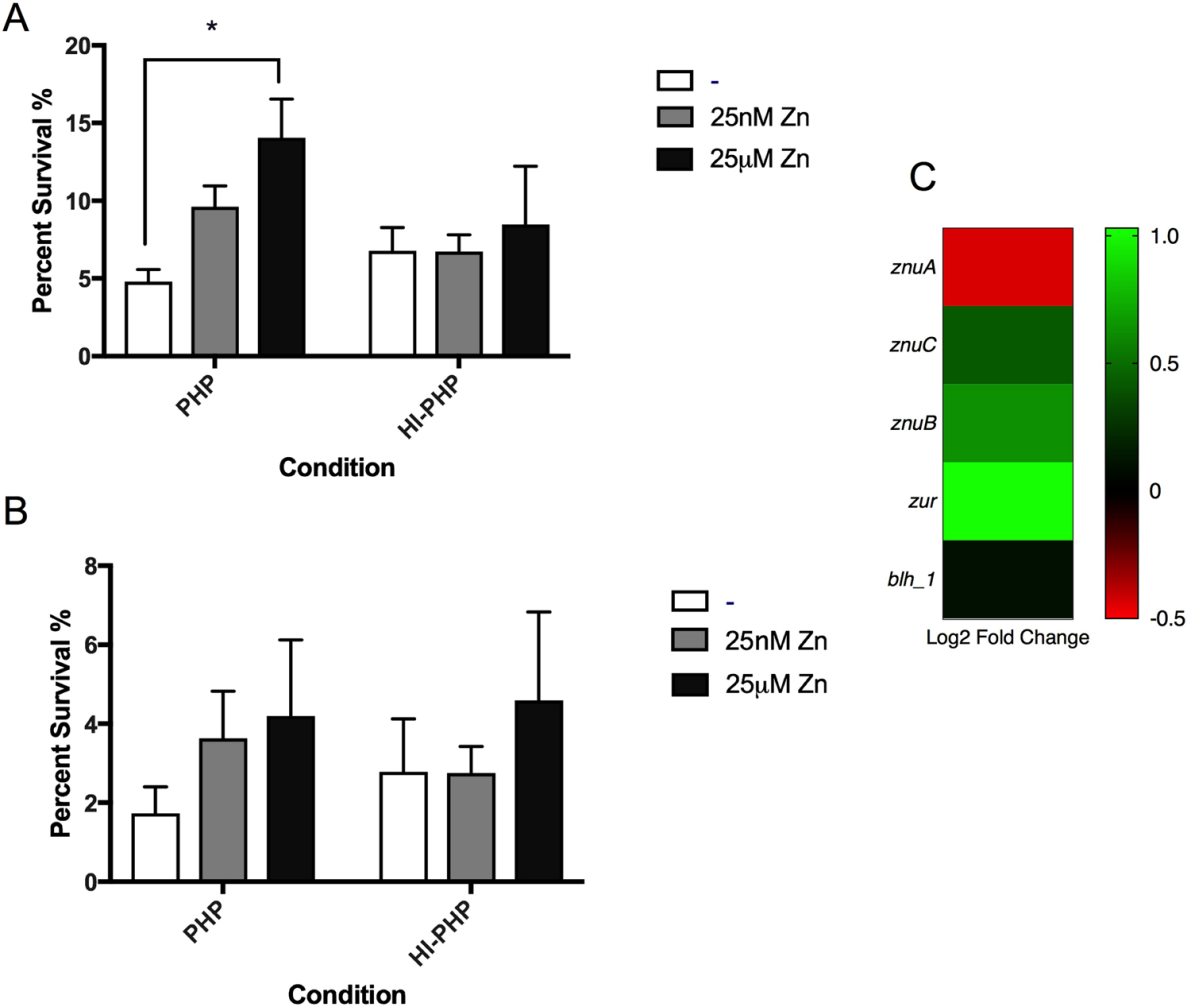
Phenotypic and genetic analysis of zinc associated genes. (**A**) Survival assays at 90 minutes performed in pooled human plasma (PHP) and heat-inactivated pooled human plasma (HI-PHP) shows that percent survival statistically significantly increases with higher concentrations of zinc in PHP. (**B**) Survival assays at 120 minutes show an increase in survival in PHP with the addition of zinc. (**C**) A heatmap outlining the differential expression of 5 genes associated with zinc survival shows four up regulated (green) genes and one down regulated genes (red).

Next, we decided to explore *A. baumannii* persistence during infection. Considering that global transcriptional regulators, such as the previously described *yebC and h-ns*, have a role in the expression of virulence features, plasma survival assays were conducted. The YebC protein family, has been shown to act as negative transcriptional regulator of proteolytic genes in both *P. aeruginosa* and *Lactobacillus*^36,45^. In fact, the derepression of YebC in the *Lactobacillus* proteolytic system plays a key role in furthering of bacterial growth under nutrient depleting conditions^45^. The function of *yebC* has yet to be characterized in *A. baumannii*; however, considering the global transcriptional regulators role in expression of virulence features and the association of YebC with proteolytic systems, we hypothesized that a protease involved in immune clearance is secreted under HSA induction. Furthermore, immune defense against *A. baumannii* has been shown in murine lung models to rely on neutrophil-secreted calprotectin, a zinc chelator, to inhibit growth^44^. Therefore, we speculated the utilization of zinc in the actions of a potentially YebC regulated protease. Thus, plasma survival assays were performed in the presence of 25 nM and 25uM zinc, respectively, to quantify the potential delay of immune clearance. Supplementing plasma samples with 25 nM zinc resulted in a 2.01-fold increase in survival at 90 minutes (*P*-value = 0.01) and a 2.10-fold increase in survival at 120 minutes (*P*-value = 0.14) (Fig. 4A and B). Inactivation of heat-labile plasma proteins resulted in an increase of *A. baumannii* survival at both time points, and addition of zinc in heat-inactivated plasma was not statistically significant. Therefore, the statistically significant increase in survival rate under zinc supplementation at 90 minutes in plasma (Fig. 4A) signifies the impact zinc availability has on *A. baumannii* survival in the presence of active humoral plasma proteins. A similar trend was observed at 120 minutes (Fig. 4B); however, the reported values were not statistically significant.

Additionally, concentrating zinc to 25uM culminated in an even higher rate of survivability, with a 2.93-fold increase in survival at 90 minutes (*P*-value = 0.003) and a 2.43-fold increase at 120 minutes (*P*-value = 0.103) (Fig. 4A and B). For both time points, a general trend of increased survival with augmented zinc supplementation was observed, indicating a positive correlation between zinc availability and *A. baumannii* survival in human plasma.

In addition, we found a zinc dependent hydrolase encoded by *blh_1* that was slightly up regulated (see Supplementary Table S2). Bacterial hydrolases that use zinc as a co-factor have been shown to be an important part of pathogenesis, encoding different metalloproteases and ß-lactamases that respond in various survival-threatening conditions, including: ß-lactam presence, host barriers, neutrophil secretion, cytokine/interleukin signaling, immunoglobulin action, and other immunological responses to pathogen invasion^46^.

Avoiding immune clearance is an important strategy for survival during bacterial pathogenesis, and human plasma is an excellent candidate for characterizing this immune clearance due to the various humoral immune proteins present. The main proteins making up 7% of human plasma include HSA, globulins, fibrinogen, and other regulatory proteins^47^. Furthermore, increasing zinc availability in plasma survival assays of *A. baumannii*, results in increased survival, suggesting that a persistence mechanism exists to inhibit host coagulation cascades and/or reduce the activity of resident plasma proteins involved in immune clearance by zinc-dependent hydrolases. This is further corroborated by the up regulation of zinc transporters *znuB* and *znuC*, as well as the down regulation of the *znu* operon transcriptional repressor *zur* (see Supplementary Table S2). This zinc availability strategy may be a persistence mechanism exhibited by *A. baumannii* as the co-factor could be shuttled to zinc-dependent hydrolases that play a role in survival in the host environment. Further studies using *in-vivo* models are necessary to explore the relationship between zinc availability and *A. baumannii* survival, as well as to illuminate which plasma proteins the potential zinc-hydrolase targets.

### HSA can have a role in the expression of genes related with resistance to ß-lactams

**Figure 5:**
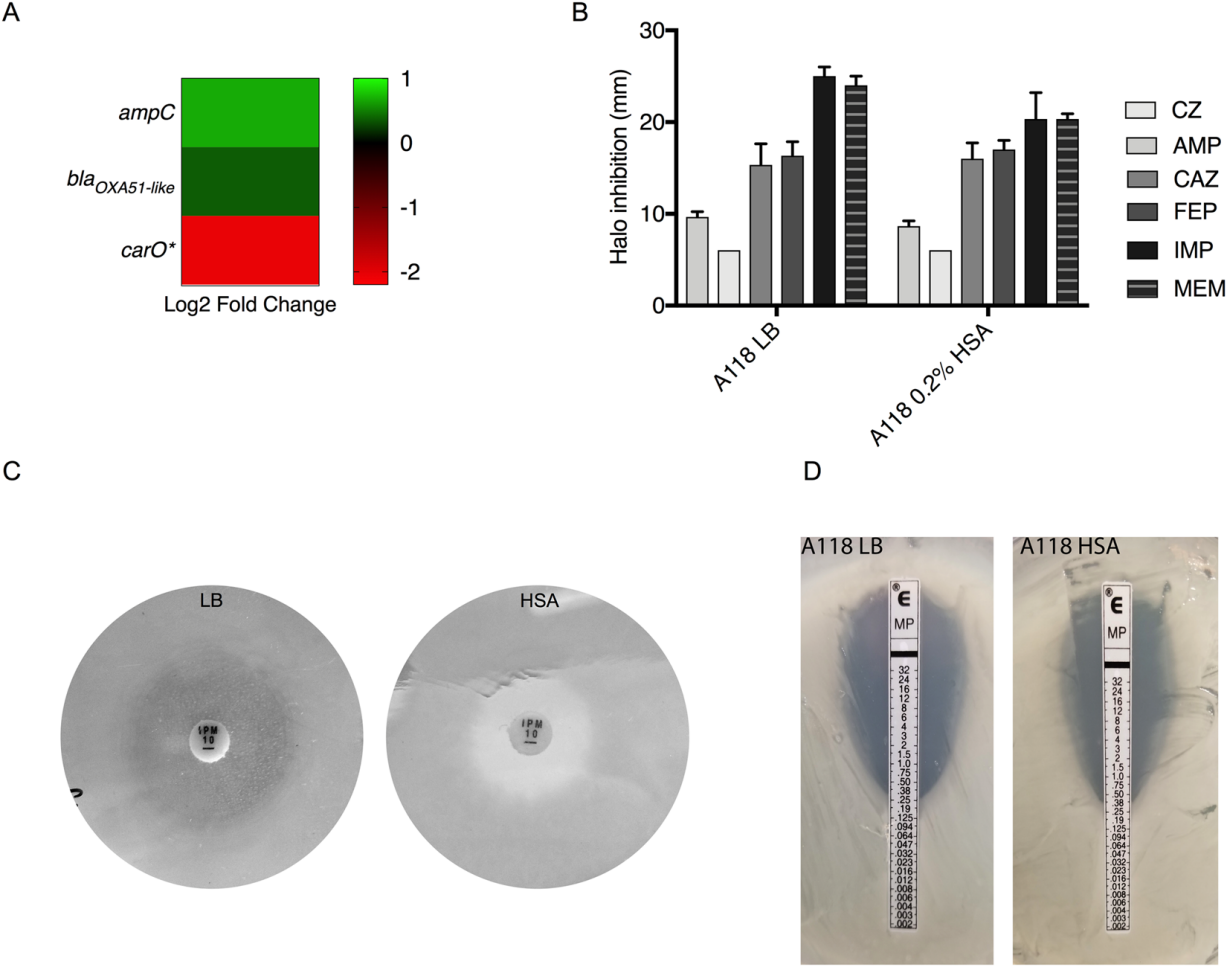
Phenotypic and genetic analysis of ß-lactam genes. (**A**) A heatmap of three ß-lactam associated genes shows the down regulation (red) of *carO* and the up regulation (green) of *ampC* and bla_OXA51-like._ (**B**) Disk-diffusion assays were performed and changes in the halo of inhibition were recorded for cefazolin (CZ), ampicillin (AMP), ceftazidime (CAZ), cefepime (FEP), imipenem (IMP) and meropenem (MEM). The photographs depict the results of (**C**) an imipenem (IPM) disk diffusion assay and (**D**) a meropenem (MP) E-test assay for A118 cells cultured with and without HSA.

*A. baumannii* is known to express a wide range of antimicrobial resistance factors, but in this particular case, multiple ß-lactam resistance mechanisms were specifically differentially expressed in the presence of HSA. Among some of the genes related with ß-lactam resistance, the gene that encodes for the outer membrane protein CarO was significantly down regulated (log_2_ fold change = −2.05). It is known that the loss of CarO is related with carbapenem-resistance^48^. Considering this and the fact that the two ß-lactamases (*bla*_ADC_ and *bla*_OXA-51-like_) found within the A118 genome were up regulated (log_2_ fold change = 0.67 and 0.36, respectively) (Fig. 5A) under HSA induction, susceptibility assays were performed. A non-statistically significant decrease on the halo of inhibition was observed for the two carbapenems tested (imipenem, IMP; and meropenem, MEM) under HSA condition (Fig. 5B and C). Changes were not observed for ampicillin, cefazolin, ceftazidime and cefepime. A notable decrease in the halo of inhibition for IMP was observed and is shown in Fig. 5C. Minimum inhibitory concentration (MIC) for MEM was also performed and a fold change increase of 0.65 in the MIC was observed (Fig. 5D). This increase in resistance addresses the impact of HSA on the fitness of *A. baumannii*, as intrinsic upregulation of ß-lactamases and down regulation of the CarO porin will lead to better survival of the microbe upon introduction of carbapenem antibiotics.

Other antibiotics also effective for *A. baumannii* treatment, such as gentamicin, amikacin, tigecycline, minocycline and polymyxin B, were tested. No changes were observed in the halo of inhibition in the conditions tested.

### HSA can influence expression of genes related with metabolism

**Figure 6:**
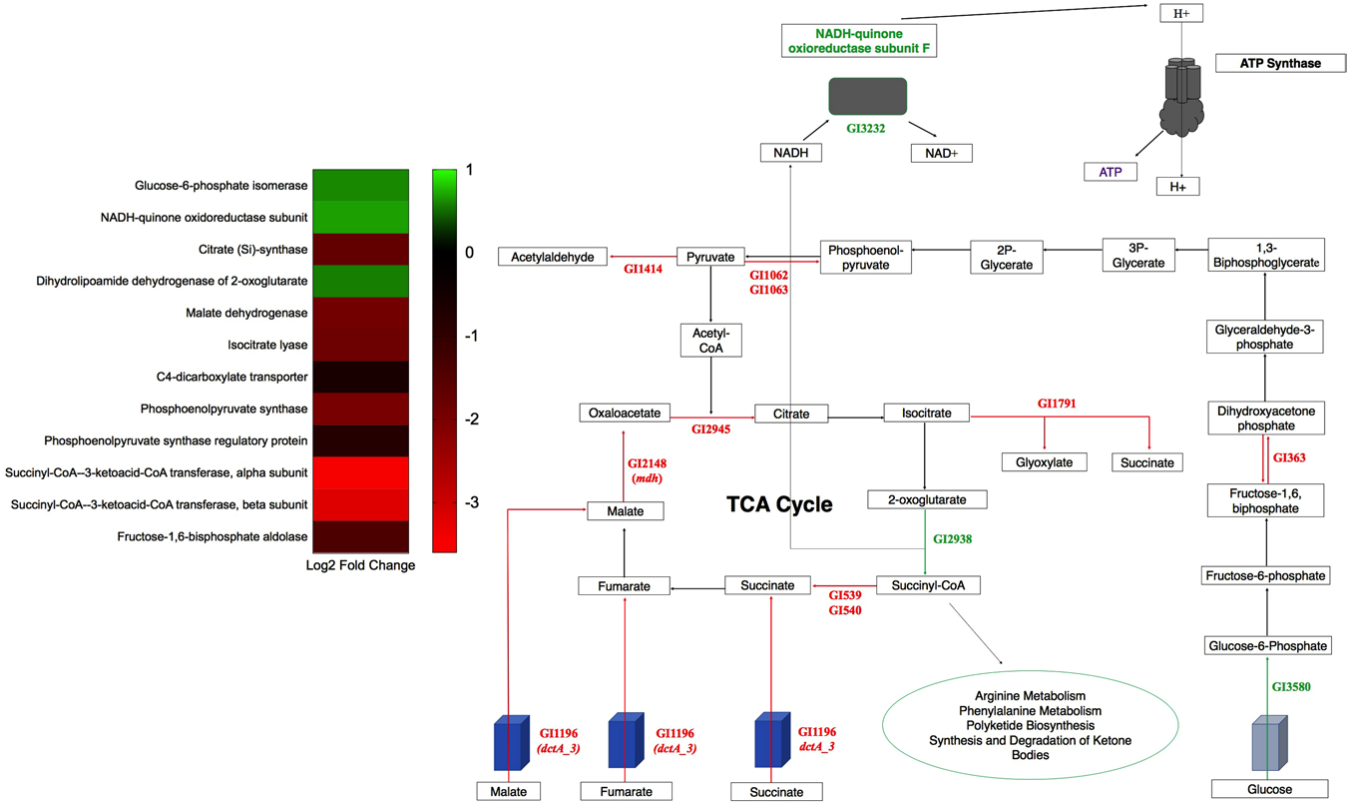
Central carbon metabolism schematic. Genes involved in central carbon metabolism that were statistically differentially expressed under HSA treatment. Words or arrows in red represent statistically significant genes that were down regulated. Words or arrows in green represent statistically significant genes that were up regulated. A heatmap of central carbon metabolism statistically differentially expressed genes is also shown.

Transcriptome analysis revealed sixty-one genes statistically significant and differentially expressed under HSA treatment involved in metabolism and nutrient acquisition (see Supplementary Table S1 and Fig. 6). Of these sixty-one genes, fifty-five were down regulated and six were up regulated. Two of these genes (*dctA* and *mdh*) have direct links to central metabolism which is important for energy production, cell synthesis, and overall growth capabilities.

**Figure 7:**
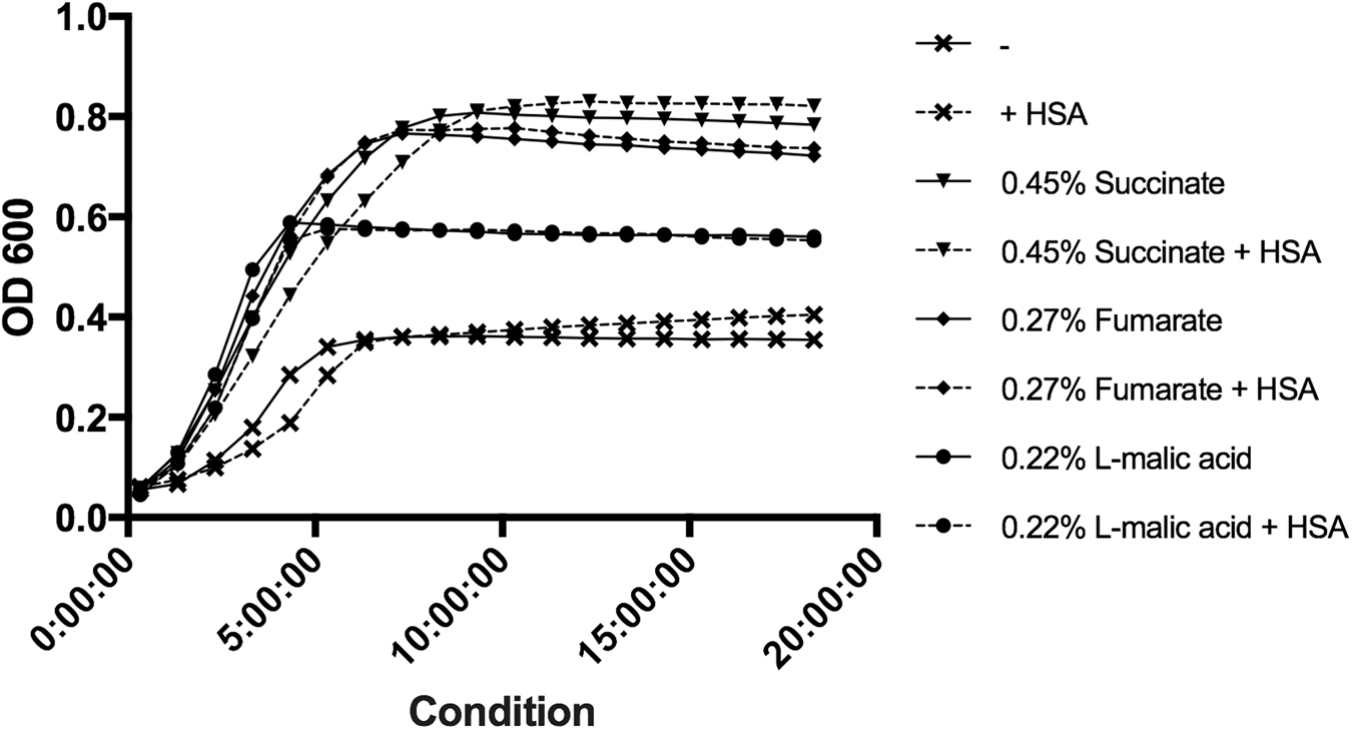
Phenotypic analysis of C_4_- dicarboxylate acquisition gene. Growth curves of *A. baumannii* strain A118 in M9 liquid minimal medium supplemented with a sole carbon source of succinate, fumarate, or L-malic acid. With or without HSA. nutrient acquisition assays showed no difference in A118 growth, measured at an optical density of 600 nm, in single dicarboxylate sourced medias.

*dctA* encodes an aerobic transmembrane C_4_-dicarboxylate transport protein that plays a role in uptake of C_4_-dicarboxylates for utilization under aerobic growth conditions^49^. NCBI BLAST identified the gene product as a member of the GltP domain and a Na^+^/H^+^ dicarboxylate symporter. *dctA* was statistically significantly differentially down regulated (see Supplementary Table S1 and Fig. 6). Additionally, a second dicarboxylate transporter, GI272, was statistically significantly differentially down regulated (log_2_ fold change of −1.78). To examine the effect of HSA on acquisition of C_4_-dicarboxylates in relation to aerobic growth, nutrient acquisition assays were performed on strain A118 of *A. baumannii*. Under HSA induction, A118 demonstrated no change in growth when mediums were supplemented with various C_4_-dicarboxylate sources (Fig. 7).

In *E. coli*, aerobic conditions allow external C_4_-dicarboxylates substrates of DctA like succinate, fumarate, L-malate to be fed into the tricarboxylic acid cycle (TCA) and oxidized. In fact, deletion of *dctA* led to reduction in succinate transport and abolition of fumarate transport, whereas phenotypic assays showed that the gene was required for aerobic growth on fumarate, L-malate, and succinate^50^. Reducing the amount of extracellular succinate, fumarate, and L-malate shuttled into *A. baumannii* due to the presence of HSA may be a potential persistence strategy. Availability of free dicarboxylates during infection may be limited, therefore down regulation of the transmembrane transporter can focus microbial energy on other forms of metabolic pathways. Therefore, the nutritional acquisition assays conducted (Fig. 7) should not demonstrate reduced growth patterns under HSA treatment, as free dicarboxylates were readily available.

**Figure 8:**
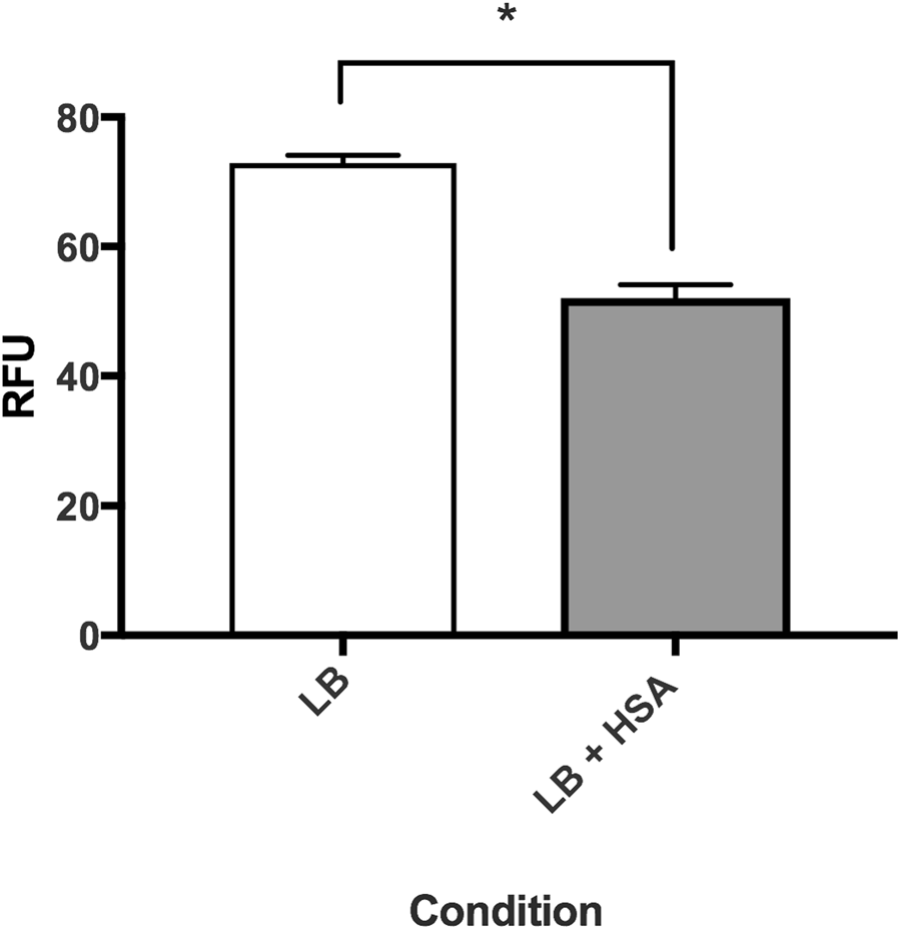
Phenotypic analysis of malate dehydrogenase activity. Malic enzymatic assays show that soluble extracts of *A. baumannii* strain A118 grown in LB, with or without HSA, have statistically significant reduced NADH formation under HSA induction. NADH formation, an indicator of malic dehydrogenase activity, was detected by relative fluorescence units (RFU) at an excitation spectrum of 340 nm and an emission spectrum of 460 nm.

A second metabolism associated gene found to be statistically significant down regulation was *mdh* (log_2_ fold change of −1.93) (see Supplementary Table S1), upon HSA induction. NAD-linked malate dehydrogenase is an oxidoreductase which catalyzes the conversion of malate into oxaloacetate using NAD as a cofactor and plays an essential role in the TCA cycle. In *E. coli*, MDH is known to be highly regulated to adapt to changing conditions such as aerobic and anaerobic cell growth and is also known to be involved in biofilm growth^51^. To phenotypically characterize the down regulation of *mdh* by HSA, lysozyme derived cell soluble extracts were assayed for NAD^+^-dependent malate dehydrogenase activity by spectrophotometric analysis of NADH formation. Malate dehydrogenase activity assays induced by HSA demonstrated a 1.47-fold decrease in NADH formation (*P*-value < 0.0001) when supplemented with malate (Fig. 8). Moreover, the reduction of biofilm production (Fig. 2A) may be linked to the reduction in malate dehydrogenase activity under HSA induction. This is further confirmed by the correlation of high biofilm forming ability of *A. baumannii* strain 1656-2 with the up regulation of malate dehydrogenase^52^.

Along with malate dehydrogenase (GI12148), four TCA cycle associated enzymes were statistically significantly differentially expressed under HSA induction: one up regulated and three down regulated. The gene encoding the dihydrolipoamide dehydrogenase subunit of 2-oxoglutarate dehydrogenase complex, which catalyzes the reduction of 2-oxoglutarate to succinyl-CoA (Fig. 6), was statistically significantly up regulated (see Supplementary Table S1). Additionally, the subsequent genes in the cycle that encode the enzymes responsible for the synthesis of succinate from succinyl-CoA (GI539 and GI540), were statistically significantly down regulated. The third gene that was also statistically significantly down regulated is the gene GI1791, which encodes for the isocitrate lyase. This enzyme is known to compete with the isocitrate dehydrogenase (IDH) for isocitrate usage outside the TCA cycle. The differential expression of these four genes suggests that succinyl-CoA production is a favored pathway in *A. baumannii* in the presence of HSA. Moreover, we also observed a statistically significant up regulation of *nuoF*, GI3232, which encodes subunit F of NADH-ubiquinone oxidoreductase (see Supplementary Table S1). NADH-ubiquinone oxidoreductase shuttles electrons to quinones in the respiratory chain to create the proton gradient along the membrane that is coupled to energy production (Fig. 6). In gram-negative bacterium, inhibition of this vital respiratory enzyme, by the action of polymyxins, results in rapid cell death^53^. The up regulation of *nuoF* (GI3232), coupled with the fact that GI2938 was the only NADH producing enzyme suggests a survival strategy implemented by *A. baumannii* under HSA induction to focus TCA flux on succinyl-coA/NADH formation.

Succinyl-coA is a precursor molecule for arginine metabolism, phenylalanine metabolism, synthesis and degradation of ketone bodies, and polyketide biosynthesis. Interestingly, *h-ns*, the global transcriptional regulator mentioned above, was linked with polyketide biosynthesis^34^. As stated before, *h-ns* was statistically down regulated upon HSA induction; therefore, we propose a potential persistence strategy associated with polyketide biosynthesis in *A. baumannii* under HSA induction. Polyketide biosynthesis enzymes are involved in the production of secondary metabolites that precede the synthesis of natural products implicated in *A. baumannii’s* motility, quorum sensing, and biofilm production^54^. This demonstrates *A. baumannii’s* pathoadaptability as it favors alternative metabolic pathways that prompt phenotypic switches to promote persistence in the human host.

Another gene statistically significant and differentially expressed, and associated with succinyl-coA production, that called our attention was *paaA*. This gene is part of the *paa* operon involved in aromatics compounds degradation to form acetyl- and succinyl-CoA later used in the TCA cycle^55^. PAA is involved in the phenylacetic acid (PA) catabolic pathway, a virulence associated pathway in *A. baumannii*. Bhuiyan *et al*. 2016, showed that inhibition of the pathway decreases the severity of the infection in a zebrafish model^56^. In *Burkholderia cepacia* the mutation of *paaA* and *paaE* drastically reduce growth on PA and decrease the virulence of this pathogen^57^.

In *A. baumannii*, transcriptomic analysis followed by phenotypic confirmation, showed an increase of the degradation of PA when *A. baumannii* grew in the presence of mucin^11^. In line with our results, Muller *et al*. 2017, showed that blue light inhibited the PA degradation pathway coding genes^14^.

**Figure 9:**
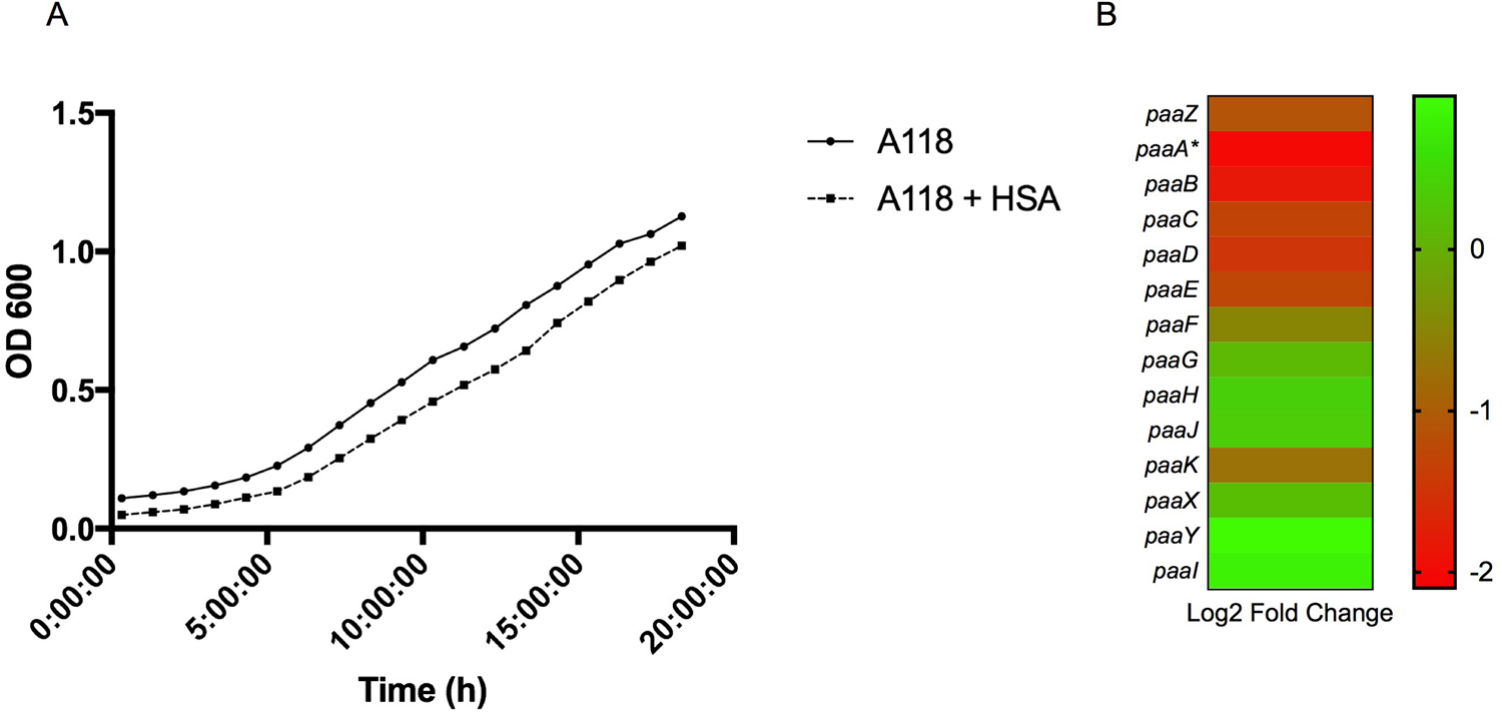
Phenotypic and genetic analysis of phenylalanine degradation pathway. (**A**) Growth curves of *A. baumannii* strain A118 in M9 liquid minimal medium supplemented with 0.2% PA as a sole carbon source. (**B**) A heatmap outlining the differential expression of genes involved in the phenylacetic acid (PA) catabolic pathway.

To confirm the effect of HSA in the PA catabolic pathway, a growth curve for PA as a sole carbon source in 0.2% PA was performed and a significant decrease (*P*-value < 0.05) in growth was observed under HSA condition (Fig. 9A). In addition, our transcriptomic analysis showed that eight out of the fourteen genes of the *paa* operon are down regulated (Fig. 9B and see Supplementary Table S2). Among the genes that are up regulated, the repressor *paaX* and *paaY* were found. The repression of the expression of this operon could decrease *A. baumannii* virulence allowing it to persist more within the host.

Another possibility is that HSA induction favors other pathways associated with succinyl-coA production, like polyketide biosynthesis, mentioned above, and arginine metabolism. In fact, the first enzyme involved in the L-arginine degradation pathway, encoded by *astA*, was statistically significantly up regulated in the presence of HSA. *astA* encodes arginine-N-succinyltransferase, an enzyme that catalyzes the production of CoA and N_2_-succinyl-L-argnine from succinyl-coA and L-arginine. Our transcriptome analysis revealed that three of the five genes of the *astCADBE* operon that act in the L-arginine degradation pathway were up regulated (GI2256-GI2260; see Supplementary Tables S1 and S2). In *E. coli*, the L-arginine degradation pathway (AST) is responsible for use of arginine as a sole nitrogen source during exponential growth^58^ Therefore, up regulation of arginine catabolism under HSA reduction may be a metabolic persistence strategy implemented by *A. baumannii*.

### Other genes of interest than can contribute to *A. baumannii* pathogenesis

Access to metal ions, such as zinc and iron, is essential for *A. baumannii*’s survival, as these ions are necessary for many cellular processes to proceed^38^. Many hosts employ a strategy that involves the depletion of these essential ions to limit the expansion and survival of pathogens and in response, *A. baumannii* has developed a mechanism to more efficiently sequester and take up iron and zinc^38^.

**Figure 10:**
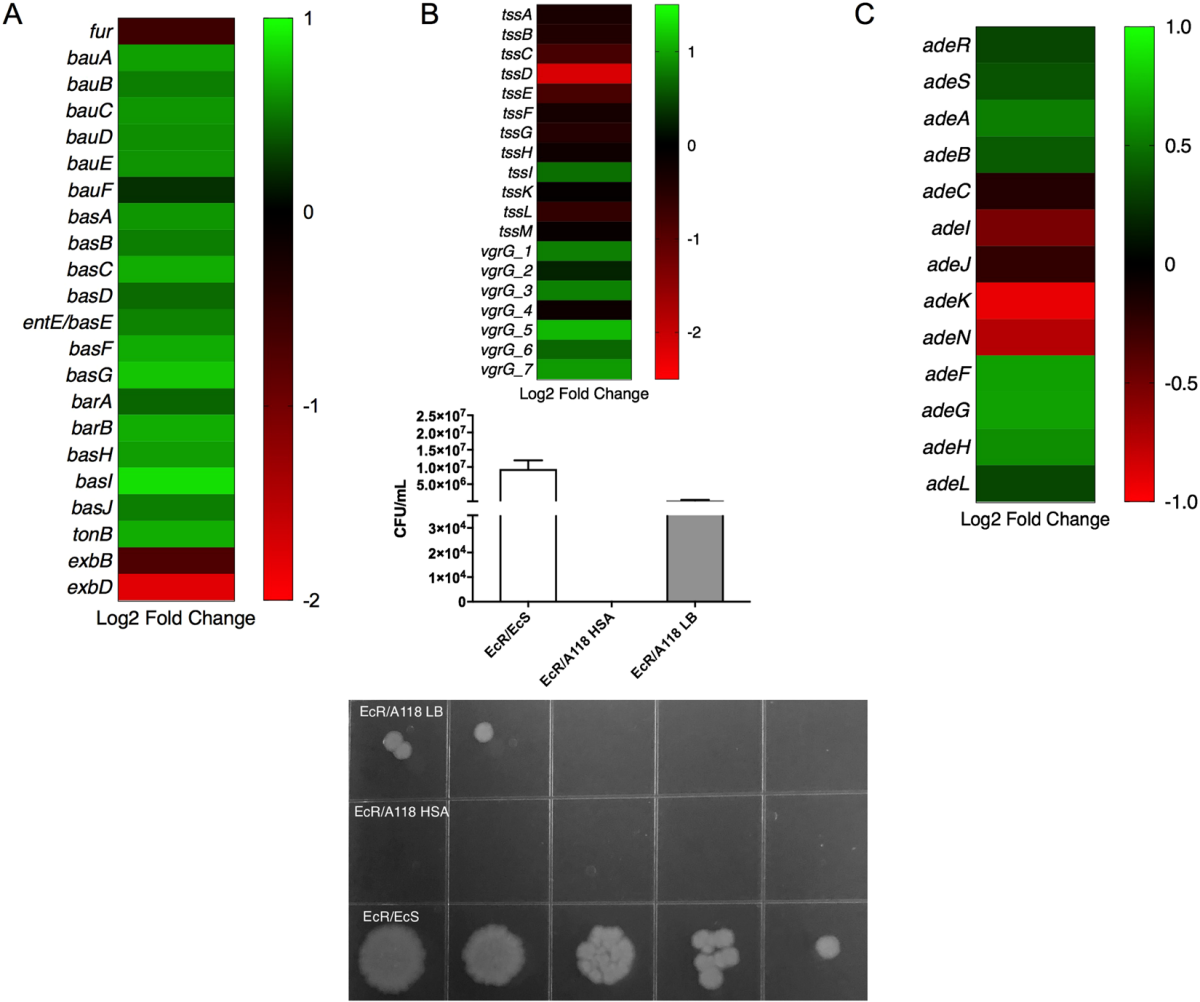
Phenotypic and genetic analysis of additional genes of interest under HSA treatment. (**A**) A heatmap outlining the differential expression of 22 iron associated genes. (**B**) A heatmap outlining the differential expression of 12 type VI secretion system genes. The associated graph depicts the difference in survival of bacterial colonies (*E. coli* MG1655-Rif- prey) with and without HSA. Representative LB-rifampicin plates are included and correspond with the graph. (**C**) A heatmap outlining the differential expression of 13 RND-type efflux pump genes.

Fur, the ferric uptake regulator was also down regulated under HSA induction (log_2_ fold change = −0.58). Genes associated with Fur potentially include the acinetobactin genes and *tonB-exbB-exbD* system^38^. Of nineteen genes identified as being involved in the biosynthesis and transport of acinetobactin in *A. baumannii*, all of them are were up regulated under HSA^59^ (Fig. 10A and see Supplementary Table S2).

The Type VI secretion system (T6SS) is a complex secretion system found in a number of Gram-negative bacteria and has been shown to play a role in the targeting eukaryotic cells and competitor bacterial cells^60^. As this system is an important virulence factor in *A. baumannii* and contributes substantially to its success as a pathogen, we examined the expression of T6SS associated genes in our transcriptomic analysis^60^. Of the T6SS core proteins (*tssA-M)*, our transcriptomic analysis revealed that all the genes were down regulated being *tssD* statistically significant down regulated with a log_2_ fold change of −2.16 (Fig. 10B and see Supplementary Table S1). Some of the *tss* genes cluster have been characterized in more detail. For example, in *A. baumannii*, TssB plays an important role in bacterium’s competing ability^61^. According to our result, *tssB* gene is down regulated in presence of HSA, therefore, HSA could decrease the bacterial competitor activity. Moreover, the *tssM* gene was reported as suppressor of host innate immune response^62^ and *tssD*, which is also known as *hcp*, can be an additional contributing gene that could be playing a role in persistence^63^.

Interestingly, the seven *vgrG* genes were identified, of which six were up regulated and only one (*vgrG_4*) was down regulated (Fig. 10B and see Supplementary Table S2)^64^. The role of *vgrG* in *A. baumannii* was related with rate growth, virulence and antimicrobial susceptibility, however, it is not implicated in biofilm formation^64^. The authors observed that ATCC 19606*ΔvgrG* exhibited reduced resistance to chloramphenicol (>256 to 96 μg/mL) comparing with the isogenic strain^64^. The observed up regulation in presence of HSA could suggest an increase in chloramphenicol resistance. In order to assess this, antimicrobial susceptibility testing in presence and absence of HSA were performed and we observed only one-fold increase in the MIC of chloramphenicol resistance (5 μg/mL to 10 μg/ml with HSA).

Our results are in agreement with previous observation where not all the core genes of the T6SS are equally expressed^11,65^. Bacterial killing assays were performed to study the biological effect of HSA. A118 and A118 under HSA treatment were used as predator, and *E. coli* MG1655-Rif was used as prey^11,65^. We observed that A118 kills *E. coli* MG1655-Rif under both conditions, and a fold change increase of 240,000 in killing activity was observed in the presence of HSA when compared to the LB condition. This results is in agreement with our transcriptomic analysis (Fig. 10B).

*A. baumannii* contains three RND-type efflux pumps that play an important role in its survival in a host and resistance to antimicrobials^66,67^. AdeABC, which is known to increase resistance to aminoglycosides, tetracyclines, fluoroquinolones, trimethoprim, chloramphenicol, ß-lactams, and tigecycline, is regulated by the two-component system AdeRS^66^. AdeFGH, which also confers resistance to chloramphenicol, fluoroquinolones, trimethoprim, tetracycline and tigecycline as well as clindamycin and sulfonamide, is regulated by the LysR-type transcriptional regulator, AdeL^66^. AdeIJK, which contributes to resistance to ß-lactams, chloramphenicol, tetracyclines, erythromycin, fluoroquinolones, fusidic acid, novobiocin and trimethoprim, is regulated by the TetR transcriptional regulator AdeN^66^.

Our transcriptomic analysis results revealed that *adeAB* and its regulator, *adeRS*, were up regulated under HSA induction (Fig. 10C and see Supplementary Table S2). Genes *adeAB* and *adeRS* have been known to be associated with antibiotic resistance, biofilm formation and virulence from previous studies. Studies from Richmond *et al*. 2016, have shown the deletion of these two genes resulted in an increase of antibiotic susceptibly, decreased biofilm formation in abiotic and biotic surfaces, and a decrease in virulence within *A. baumannii*^30^. Also, transcriptomic analysis of *adeFGH* revealed that the coding genes of this efflux pump are up regulated (Fig. 10B and see Supplementary Table S2). In contrast, the AdeIJK efflux pump genes were down regulated, and the associated transcriptional regulator, *adeN*, was also down regulated (Fig. 10B and see Supplementary Table S2).

Several bacterial species use the T6SS and/or efflux pumps during biofilm formation^45,67,68^. Mutations in the enteroaggregative *E. coli* Sci-1 T6SS or avian pathogenic *E. coli* T6SS genes decrease the ability to form biofilms^45^. This is in line with our biofilm results, where a decrease in biofilm formation in presence of HSA was observed. The down regulation of *tssA-M* genes cluster, the *adeIJK* operon and its *adeN* regulator in presence of HSA could also explain the decrease of biofilm formation. Thus, *A. baumannii* could be attenuated its virulence in presence of an HSA.

In summary, considering the data obtained from the transcriptomic analysis of *A. baumannii* (strain A118) under HSA treatment, different relevant phenotypes associated with the pathoadaptability and pathogenesis of this pathogen were studied. We identified how this essential human protein can play a role in the survival and persistence of this pathogen. Among the 296 protein-coding genes that showed statistically significant differential expression, we focused on genes associated with motility, biofilm formation, antibiotic resistance, virulence, metabolic processes, and transcriptional regulation. Phenotypic analysis of these traits under HSA induction revealed an increase in surface-associated motility, a decrease in biofilm formation, a slight increase in antibiotic resistance against ß-lactam antibiotics, and a decrease in malate dehydrogenase activity.

*A. baumannii* infections are associated with attributable mortality levels as high as 60% (through community-acquired pneumonia) and 43.4% (through bloodstream infections)^1–3^. This high rate can be explained in part by the acquisition and expression of resistance genes, the ability to produce capsule, biofilm, and the possibility to activate different metabolic and acquisition pathways^69^. Here we observed that HSA has a noticeable effect on traits that can help *A. baumannii* adapt to environmental changes, and can contribute to pathogenicity. The observed increase in motility, together with the increase rate in survivability, its’ ability to switch metabolism, as well as, as increase antibiotic resistance and virulence under HSA induction, can contribute to the success of *A. baumannii* using a multifactorial approach. We acknowledge that our study has limitations. The study was performed *in vitro* in isolation using only one *A. baumannii* strain. Despite these limitations, both the differential genotype and phenotype effects under HSA treatment were conclusive and are relevant in the pathogenesis of this species. We consider this to be solid data that can open new venues for future *in vivo* studies. Further studies can help uncover *A*. *baumannii* mechanisms of survival and persistence within the host, as well as, explain the utilization of albumin in *A. baumannii* success as a nosocomial pathogen.

## Material and Methods

### Bacterial Strains

*A. baumannii* strain A118 was used for RNA and phenotypic analysis. The strain was isolated from the blood sample of a hospitalized patient in Argentina, has had its genome sequenced and has been shown to be naturally competent and susceptible to a variety of antibiotics (Ramirez, 2010; Traglia, 2014). Cells were initially plated from

80 °C storage on Cystine lactose electrolyte deficient (CLED) agar to obtain fresh cells and later cultured in Luria Bertani (LB) broth at 37 °C.

### RNA Extraction

A118 cells were cultured in Luria Bertani broth (LB) with or without 0.2% human serum albumin (HSA) and incubated with agitation for 18 h at 37 °C. Overnight cultures were then diluted 1:10 in fresh LB broth and incubated with agitation for 7 h at 37 °C. RNA was immediately extracted following the TRI REAGENT® Kit (Molecular Research Center, Inc., Cincinnati, Ohio, USA). Briefly, cell pellets were lysed in TRI REAGENT® and incubated at 23 °C for 5 m. Chloroform was added and samples were incubated at 23 °C for 15 m and then centrifuged at 13,200 rotations per minute (rpm) at 4 °C for 15 m to induce phase separation. The aqueous layer was transferred to a tube containing isopropanol, incubated at room temperature for 10 m and centrifuged at 13,200 rpm at 4 °C for 8 m. The RNA pellet was washed with 70% ethanol and allowed to air dry, before being dissolved in RNase-free water. Quantification of RNA was performed using a DeNovix DS-11+ Spectrophotometer and qualification was assessed on a 1.2% agarose gel via gel electrophoresis. DNase treatment was performed following the manufacturer’s instructions (Thermo Fisher Scientific, Waltham, MA, USA) and results were quantified as previously described. Samples were confirmed to have no DNA contamination through PCR amplification of the 16S rDNA gene as follows: an initial denaturing stage of 95 °C for 5 m, a three-step reaction of 95 °C for 1 m, 54 °C for 30 s and 72 °C for 2 m, repeated 28 times, and a final stage of 72 °C for 5 m. PCR products were visualized on a 1% agarose gel and genomic DNA extracted from A118 was used as a positive control. Extractions were performed in triplicate and control and experimental were performed in parallel.

### RNA Sequencing and Analysis

RNA sequencing was outsourced to Otogenetics (Otogenetics Corporation, Atlanta, GA) where they performed ribosomal RNA-depletion using the Ribo-Zero kit (Illumina). Construction of the cDNA library was performed with the TruSeq Stranded Total RNA Library Prep kit (Illumina) from three independent replicates per sample. Data collected generated an average of 19.5 million paired-ends reads per sample with an average of 19.8 and 18.8 total reads for LB (5 replicates) and LB + HAS (3 replicates), respectively. 3′-end adapter contaminant trimming was done using scythe (available at github.com/vsbuffalo/scythe). Then, reads were trimmed based on quality using sickle software (available at github.com/najoshi/sickle), a phred score of 35 was used as threshold. Reads were mapped against the annotated draft genome of A118 with the function “align” from the Rsubread package^70^. Mapped reads (averaged 90%) were counted based on the available annotation data using the function “featureCounts”, also from the Rsubread package^70^. Finally, differential expression analysis was carried out with the DESeq. 2 package^71^. Genes with a FDR adjusted *P*-value of <0.05 and <0.1 were considered as statistically significantly differentially expressed and subject to further analysis by Artemis Version 16.0.0, Blast2Go Version 4.1.5, NCBI BLAST and the Kyoto Encyclopedia of Genes and Genomes (KEGG). RNA-seq data generated as a result of this work has been deposited to SRA with the accession SRP149770.

### Real-time RT-qPCR

The cDNA was prepared using the iScript™ Reverse Transcription Supermix for RT-qPCR (BioRad, Hercules, CA, USA) per the manufacturer’s instructions and concentrations were measured with a DeNovix DS-11+ Spectrophotometer. Quantitative PCR was performed using iQ™SYBR® Green Supermix (BioRad, Hercules, CA, USA) per the manufacturer’s recommendations. Results were analyzed using the 2^−ΔΔCt^ method in which *recA* acted as the control gene and *pilQ* the experimental gene. Experiments were performed in technical quadruples and biological triplicate and statistical analysis (Mann-Whitney test) was performed using GraphPad Prism (GraphPad software, San Diego, CA, USA). A *P-*value < 0.05 was considered significant.

### Biofilm Assays with *A. baumannii* A118 cells

A118 cells were cultured in LB broth with or without 0.2% HSA and incubated with agitation for 18 h at 37 °C. Overnight cultures were centrifuged at 5,000 rpm at 4 °C for 5 and cell pellets were washed twice with 1X PBS and then re-suspended in 1X PBS. Following, the optical density at 600 nm (OD_600_) of each culture was adjusted to 0.9–1.1, vortexed and diluted 1:100 in LB broth before being plated in technical triplicate in a 96-well polystyrene micro-titer plate and being incubated at 37 °C for 24 h without agitation. The following day, the OD_600_ (ODG) was measured using a micro-plate reader (SpectraMax M3 microplate/ cuvette reader with SoftMax Pro v6 software) to determine the total biomass. Wells were emptied with a vacuum pipette, washed three times with 1X phosphate-buffered saline (PBS) and stained with 1% crystal violet (CV) for 15 m. Excess CV was removed by washing three more with 1X PBS and the biofilm associated with the CV was solubilized in ethanol acetate (80:20) for 30 m. The OD_580_ (ODB) was measured using a micro-plate reader and results were reported as the ratio of biofilm to total biomass (ODB/ODG). Experiments were performed in triplicate, statistical analysis (Mann-Whitney test) was performed using GraphPad Prism (GraphPad software, San Diego, CA, USA), and a *P-*value < 0.05 was considered significant.

### Motility Assays

Motility agar plates were made by dissolving 10 g LB broth powder (containing 5 g tryptone, 2.5 g NaCl, and 2.5 g yeast extract) and 2.5 g agarose in 1 L of de-ionized water to make 0.25% LB agarose plates. A118 cells were cultured in LB broth with or without 0.2% HSA and incubated with agitation for 18 h at 37 °C. 4 μL of the overnight culture was pipetted on to the center of the motility plate and was incubated at 37 °C for 24 h. The following day, the diameter of growth was measured and was classified as non-motile (<5 mm), intermediately motile (5–20 mm) or highly motile (>20 mm). Experiments were performed in triplicate, with at least three technical replicates per biological replicate. Statistical analysis (Mann-Whitney test) was performed using GraphPad Prism (GraphPad software, San Diego, CA, USA), and a *P-*value < 0.05 was considered significant.

### Opaque and translucent variants

To distinguish between opaque and translucent colonies the previously described protocol was followed^44^. Briefly, A118 cells with and without HSA were cultured for 18 h and then plated on agar plates composed of LB broth supplemented with 0.8% agar for 24 h. Ab5075 was used as control^43^. The colonies were observed using a stereo microscope with oblique lighting from underneath.

### Plasma survival assay

The plasma survival assay was performed in triplicate and prepared in the presence or absence of 25 nM ZnCl_2_ or 25 uM ZnCl_2_. Three separate conditions were used: LB broth, 90% pooled human plasma (in 1 × PBS), and heat-inactivated 90% pooled human plasma. All pooled human plasma samples were acquired from a certified vendor that obtains blood samples from FDA-approved facilities (Innovative Research, MI USA). Heat inactivation of 90% pooled human plasma was done at 56 °C for 30 minutes. Overnight culture was then diluted 10^6^ fold in 1× PBS, then the final dilution was diluted further 1:10 to a final volume of 100uL in each condition. Dilutions were plated on LB agar at times 0, 60, 90, 120, and 240 minutes. Percent survival was then calculated using control samples against experimental plasma samples at the various end points tested^18^.

### Susceptibility assays

Antibiotic susceptibility assays were performed following with the procedures recommended by the CLSI with slight modifications as described in Ramirez *et al*.^67^. Mueller-Hinton agar plates were inoculated with 100 µl of a culture of each tested condition (A118 or A118+ HSA) after OD adjustment. Antimicrobial commercial disks (BBL, Cockeysville, MD, USA) containing 30 mg of cefepime (FEP), 30 mg of ceftazidime (CAZ), and 10 mg of imipenem (IMP) were used, and the plates were incubated at 37 °C for 18 h. The assays were performed in triplicate. Statistical analysis (Mann-Whitney test) was performed using GraphPad Prism (GraphPad software, San Diego, CA, USA), and a *P-*value < 0.05 was considered significant.

### Nutrient Acquisition Assay

Cultures were grown in LB broth induced with or without human serum albumin overnight in 37 °C with shaking. Growth assays were conducted on 96-well plates in triplicate in Minimal Medium M9 supplemented with 1uM MgSO_4,_ 1 mM CaCl_e_ and either with 0.45% succinate, 0.27% dibasic fumarate, or 0.22% (−) L-malic acid. These concentrations were selected to ensure the same number of carbon atoms were present in each respective medium so the activity of the dicarboxylic acid protein could be effectively analyzed via reduction in growth rate^72^. In addition, 0.2% phenylacetic acid was used as a carbon source. Wells containing 294 uL of media and 6 uL of culture were incubated for 18 hours in 37 °C with medium shaking. Growth was measured at an OD_600_ every 20 minutes. Statistical analysis (Mann-Whitney test) was performed followed by multiple t-test of various time points of the growth curve.

### Malic enzymatic assay

Malate dehydrogenase activity was detected in cell soluble extracts of A118 in the presence or absence of HSA. 2 mL aliquots were pelleted and resuspended in 80 µL of resuspension Buffer (100 mM HEPES, 1 M MgCl_2_, pH 7.5). Soluble extracts were extracted via lysis of the cell after 0.8 mg of lysozyme was added and samples were incubated at 37 °C for 30 minutes. Samples were then centrifuged for 10 minutes at 4 °C at 12,000 rpm and supernatants were aliquoted. 16 µL of supernatant was added to 184 µL of forward reaction buffer (100 mM HEPES (pH 7.5), 10 mM NAD+, 100 mM (−) L-malic acid, 1 mM dithiothreitol, and 10 mM MgCl_2_) per well^73,74^. Malic enzyme assays were conducted in triplicate and evaluated in the direction of malate oxidation to oxaloacetate by assessing the production of NADH. End point absorbance was analyzed via spectrophotometric analysis (SpectraMax M3) at 30 °C with an excitation spectrum of 340 nm and an emission spectrum of 460 nm.

### Bacterial killing assays

Killing assays were carried using A118 and *E. coli* MG1655-Rif as predator and prey, respectively^11,65^. A118 or A118+ HSA, and *E. coli* MG1655 and MG1655-Rif LB cultures, were grown and were normalized to an OD_600_ of 0.5 and performed in duplicates. MG1655/MG1655-Rif mixtures were used as negative controls. A118 or A118+ HSA and MG1655-Rif were mixed at a predator-prey 1:1 ratio and 5 μl were spotted on a dry LB agar plate. After 4 h, the spot was resuspended in 1 ml PBS and 10 μl serial dilutions plated on LB agar plate with rifampicin (100 μg/ml)^75^. Growth of the prey was recorded after incubation at 37 °C for 36 h.

## Electronic supplementary material

Figure S1 and Figure S2

Table S1

Table S2
