# Supplementary material for: Human serum albumin alters specific genes that can play a role in survival and persistence in Acinetobacter baumannii
Source: Sci Rep. 2018 Oct 3;8:14741. doi: 10.1038/s41598-018-33072-z (PMC6170387; doi:10.1038/s41598-018-33072-z)
Supplement: Supplementary file 1 — Figure S1 and Figure S2 [file 41598_2018_33072_MOESM1_ESM.pdf]

Human serum albumin alters specific genes that can play a role in survival and persistence in *Acinetobacter baumannii*.

Brettni Quinn[1#], Nyah Rodman[1#], Eugenio Jara[2], Jennifer S. Fernandez[1], Jasmine Martinez[1], German M. Traglia[3], Sabrina Montaña[4], Virginia Cantera[8], Kori Place[1], Robert A. Bonomo[5,6,7], Andres Iriarte[8], and María Soledad Ramírez[1]*

[1]Center for Applied Biotechnology Studies, Department of Biological Science, College of Natural Sciences and Mathematics, California State University Fullerton, Fullerton, California, USA, [2]Área Genética, Facultad de Veterinaria, Universidad de la República, Montevideo, Uruguay, [3]Laboratorio de Bacteriología Clínica, Departamento de Bioquímica Clínica, Hospital de Clínicas José de San Martín, Facultad de Farmacia y Bioquímica, Buenos Aires, Argentina,[4]Instituto de Microbiología y Parasitología Médica (IMPaM, UBA-CONICET), Facultad de Medicina, Universidad de Buenos Aires, Argentina, [5]Medical Service and GRECC, Louis Stokes Cleveland Department of Veterans Affairs Medical Center, Cleveland, Ohio, USA, [6]Departments of Medicine, Pharmacology, Molecular Biology and Microbiology, Biochemistry, Proteomics and Bioinformatics, Case Western Reserve University School of Medicine, Cleveland, Ohio, USA, [7]CWRU-Cleveland VAMC Center for Antimicrobial Resistance and Epidemiology (Case VA CARES), Cleveland, Ohio, USA,[8]Laboratorio de Biología Computacional, Dpto. de Desarrollo Biotecnológico, Instituto de Higiene, Facultad de Medicina, UdelaR, Montevideo, Uruguay.

[#]These authors contributed equally to this work

Additional Information

Supplementary information accompanies this paper

Legends to Supplementary Figure and Tables

Figure S1. Plot of differential gene expression under HSA treatment. The $\log_2$ fold change values of all statistically significantly (FDR adjusted *P*-value of <0.1) differentially expressed genes identified during transcriptomic analysis of *A. baumannii* strain A118 cells during growth with or without HSA.

Figure S2. Gene ontology analysis of differentially expressed genes under HSA treatment. The significantly differentially expressed genes identified during transcriptomic analysis were categorized by their associated gene ontology terms and were placed into one or more of the following categories: cellular component, biological processes and/or molecular function, in an effort to identify their role/function. (A) Within the three main gene ontology categories, up-regulated genes are depicted in dark blue and down regulated genes are depicted in light blue. Furthermore, the number of genes associated with each gene ontology term within the (B) cellular components, (C) biological processes and (D) molecular function categories are depicted as a heat map.

Table S1 Information about all statistically significantly (FDR < 0.1) differentially expressed genes including gene ID, name, function, $\log_2$ fold change and FDR value. Genes highlighted in green are discussed in detail.

Table S2 Information about all differentially expressed genes including gene ID, annotation (when available), $\log_2$ fold change and FDR value.

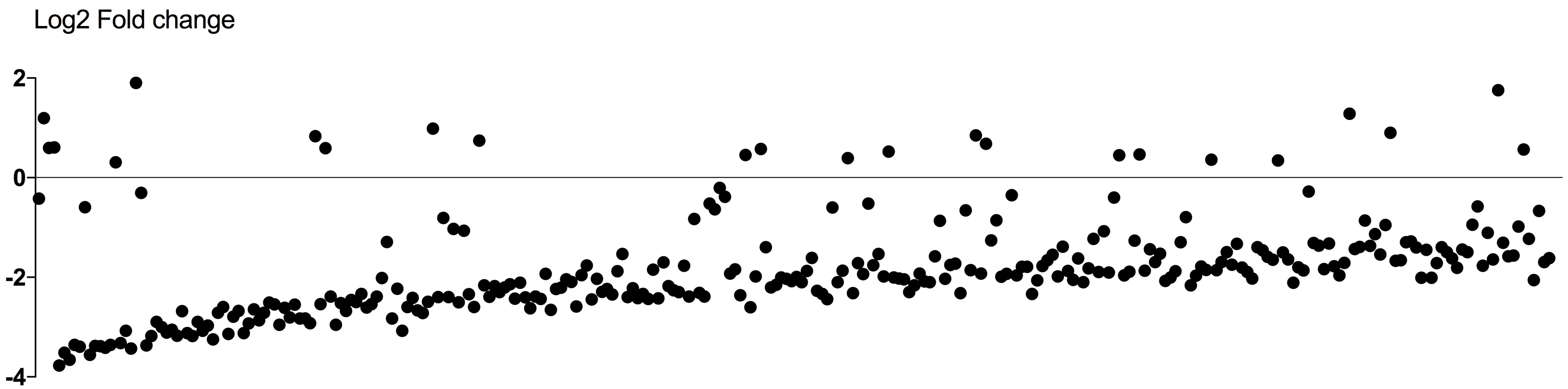

Log2 Fold change
2
0
-2
-4

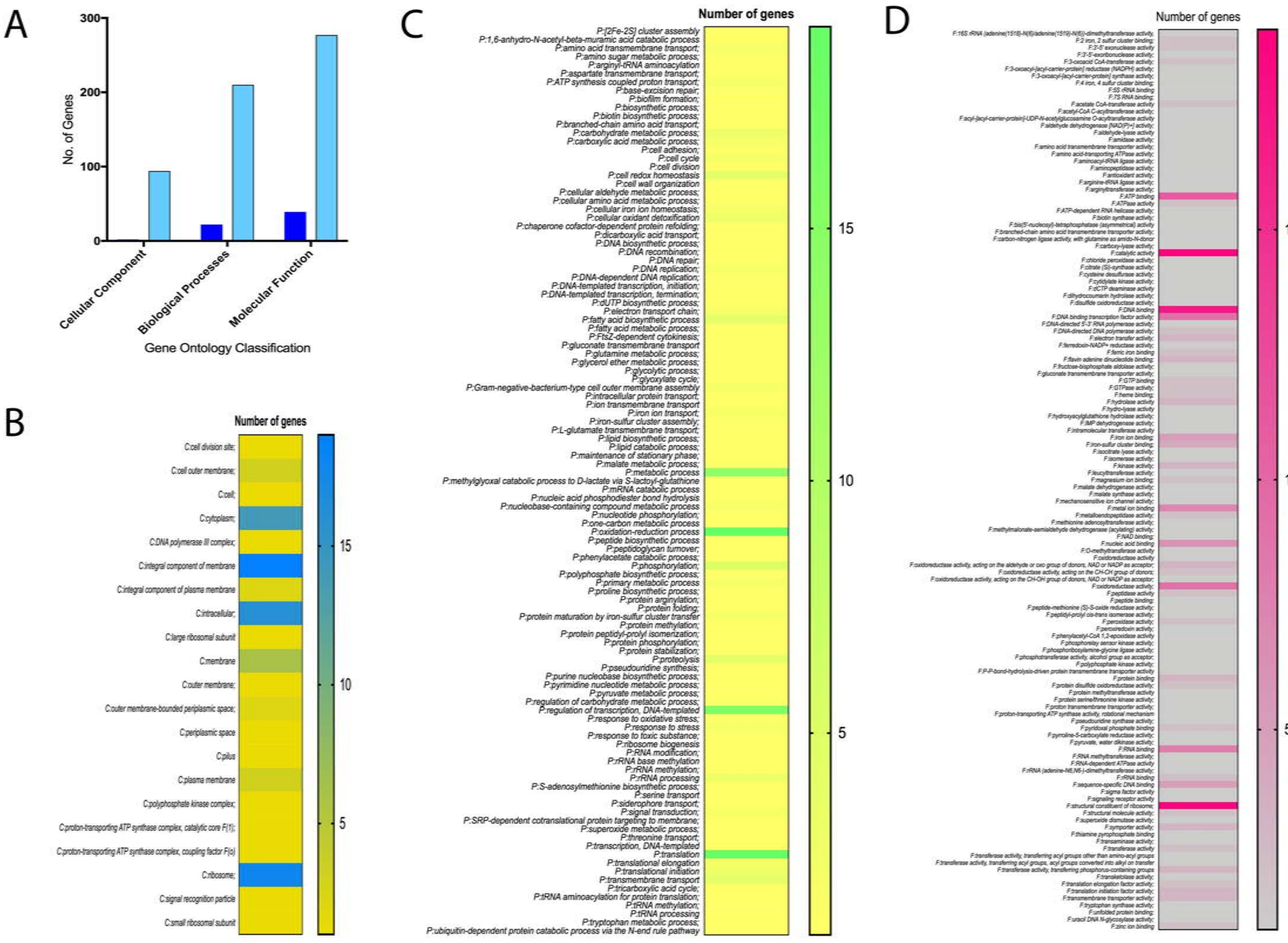
A
No. of Genes
300
200
100
0
Cellular Component
Biological Processes
Molecular Function
Gene Ontology Classification
B
Number of genes
C:cell division site;
C:cell outer membrane;
C:cell;
C:cytoplasm;
C:DNA polymerase III complex;
C:integral component of membrane
C:integral component of plasma membrane
C:intracellular;
C:large ribosomal subunit
C:membrane
C:outer membrane;
C:outer membrane-bounded periplasmic space;
C:periplasmic space
C:pilus
C:plasma membrane
C:polyphosphate kinase complex;
C:proton-transporting ATP synthase complex, catalytic core F(1);
C:proton-transporting ATP synthase complex, coupling factor F(o)
C:ribosome;
C:signal recognition particle
C:small ribosomal subunit
15
10
5
C
Number of genes
P:[2Fe-2S] cluster assembly
P:1,6-anhydro-N-acetyl-beta-muramic acid catabolic process
P:amino acid transmembrane transport;
P:amino sugar metabolic process;
P:arginyl-tRNA aminoacylation
P:aspartate transmembrane transport;
P:ATP synthesis coupled proton transport;
P:base-excision repair;
P:biofilm formation;
P:biosynthetic process;
P:biotin biosynthetic process;
P:branched-chain amino acid transport;
P:carbohydrate metabolic process;
P:carboxylic acid metabolic process;
P:cell adhesion;
P:cell cycle
P:cell division
P:cell redox homeostasis
P:cell wall organization
P:cellular aldehyde metabolic process;
P:cellular amino acid metabolic process;
P:cellular iron ion homeostasis;
P:cellular oxidant detoxification
P:chaperone cofactor-dependent protein refolding;
P:dicarboxylic acid transport;
P:DNA biosynthetic process;
P:DNA recombination;
P:DNA repair;
P:DNA replication;
P:DNA-dependent DNA replication;
P:DNA-templated transcription, initiation;
P:DNA-templated transcription, termination;
P:dUTP biosynthetic process;
P:electron transport chain;
P:fatty acid biosynthetic process
P:fatty acid metabolic process;
P:FtsZ-dependent cytokinesis;
P:gluconate transmembrane transport
P:glutamine metabolic process;
P:glycerol ether metabolic process;
P:glycolytic process;
P:glyoxylate cycle;
P:Gram-negative-bacterium-type cell outer membrane assembly
P:intracellular protein transport;
P:ion transmembrane transport
P:iron ion transport;
P:iron-sulfur cluster assembly;
P:L-glutamate transmembrane transport;
P:lipid biosynthetic process;
P:lipid catabolic process;
P:maintenance of stationary phase;
P:malate metabolic process;
P:metabolic process
P:methylglyoxal catabolic process to D-lactate via S-lactoyl-glutathione
P:mRNA catabolic process
P:nucleic acid phosphodiester bond hydrolysis
P:nucleobase-containing compound metabolic process
P:nucleotide phosphorylation;
P:one-carbon metabolic process
P:oxidation-reduction process
P:peptide biosynthetic process
P:peptidoglycan turnover;
P:phenylacetate catabolic process;
P:phosphorylation;
P:polyphosphate biosynthetic process;
P:primary metabolic process
P:proline biosynthetic process;
P:protein arginylation;
P:protein folding;
P:protein maturation by iron-sulfur cluster transfer
P:protein methylation;
P:protein peptidyl-prolyl isomerization;
P:protein phosphorylation;
P:protein stabilization;
P:proteolysis
P:pseudouridine synthesis;
P:purine nucleobase biosynthetic process;
P:pyrimidine nucleotide metabolic process;
P:pyruvate metabolic process;
P:regulation of carbohydrate metabolic process;
P:regulation of transcription, DNA-templated
P:response to oxidative stress;
P:response to stress
P:response to toxic substance;
P:ribosome biogenesis
P:RNA modification;
P:rRNA base methylation
P:rRNA methylation;
P:rRNA processing
P:S-adenosylmethionine biosynthetic process;
P:serine transport
P:siderophore transport;
P:signal transduction;
P:SRP-dependent cotranslational protein targeting to membrane;
P:superoxide metabolic process;
P:threonine transport
P:transcription, DNA-templated
P:translation
P:translational elongation
P:translational initiation
P:transmembrane transport
P:tricarboxylic acid cycle;
P:tRNA aminoacylation for protein translation;
P:tRNA methylation;
P:tRNA processing
P:tryptophan metabolic process;
P:ubiquitin-dependent protein catabolic process via the N-end rule pathway
15
10
5
D
Number of genes
15
10
5
